# Suspicion Is Key: Diagnosing Elusive Cytoplasmic Antineutrophil Cytoplasmic Antibody (c-ANCA)-Positive Vasculitis

**DOI:** 10.7759/cureus.25501

**Published:** 2022-05-30

**Authors:** Timothy P Crowe, Roberto Campos, Juan Pereira-Duque, Mohammad Samiullah, Perham Eftikari

**Affiliations:** 1 Internal Medicine, Nova Southeastern University Dr. Kiran C. Patel College of Osteopathic Medicine, Fort Lauderdale, USA; 2 Internal Medicine, Broward Health General, Fort Lauderdale, USA; 3 Nephrology, Broward Health General, Fort Lauderdale, USA

**Keywords:** antineutrophil, glomerulonephritis, vasculitis, myeloperoxidase, anca

## Abstract

We present a case of a 53-year-old black male with a past medical history of type 2 diabetes presenting with fatigue, generalized myalgias, and unintentional weight loss developed over several months. The patient was diagnosed with cytoplasmic antineutrophil cytoplasmic antibody (c-ANCA)-positive vasculitis. Renal biopsy confirmed antineutrophil cytoplasmic antibody (ANCA) crescentic necrotizing glomerulonephritis. c-ANCA was positive by enzyme-linked immunosorbent assay. Anti-myeloperoxidase antibody was positive. Without paranasal or lung involvement, the diagnosis was made of renal-limited necrotizing and crescentic glomerulonephritis. The patient was treated with rituximab and prednisone therapy.

## Introduction

Antineutrophil cytoplasmic antibodies (ANCAs) are a type of autoantibodies that are associated with necrotizing small-vessel vasculitis collectively known as ANCA-associated vasculitis (AAV). AAV includes microscopic polyangiitis (MPA), granulomatosis with polyangiitis (GPA), eosinophilic granulomatosis with polyangiitis (EGPA), and renal-limited necrotizing and crescentic glomerulonephritis (NCGN) [1]. We present a case of a 53-year-old male with a past medical history of type 2 diabetes mellitus, who presented to the emergency department with six months of fatigue, generalized myalgias, and unintentional weight loss. We will discuss the diagnosis of renal-limited ANCA glomerulonephritis and subsequent treatment.

## Case presentation

A 53-year-old Haitian male with a past medical history of type 2 diabetes initially presented with complaints of weakness and generalized myalgias for the last six months. The myalgias were felt in the chest, abdomen, lower extremities, and lower back. The patient had been following up with his primary healthcare provider for several months regarding the myalgias and was given several courses of nonsteroidal anti-inflammatory drugs, muscle relaxers, topical creams, and steroids with mild improvement. His last appointment was the morning of admission, where he was advised to go to the emergency department due to oxygen saturation in the high 70%. Upon further questioning, the patient was also found to be suffering from unintentional weight loss over the past six months and additional urinary hesitancy.

Prior to this presentation, and over the course of the previous five months, due to various ED visits for the same complaint, the patient had been treated with a combination of prednisone, naproxen, topical lidocaine, ibuprofen, and cyclobenzaprine, with minimal to no relief of symptoms. The patient was a tobacco smoker (six to seven cigarettes daily) reporting to have quit two weeks prior to the latest admission. He drank alcohol occasionally and denied illicit drug use. He was not married and worked in waste management. Family history included type 2 diabetes mellitus in his mother and sister, hypertension in his father, and stroke in his sister; moreover, there was no personal or family history of malignancy, vasculitides, bleeding, or clotting disorders. A review of systems was positive for unintentional weight loss, chest wall pain, urinary hesitancy, generalized myalgias, joint pain, back pain, and generalized weakness.

On presentation to the hospital, he was afebrile at 97.7°F, tachycardic at 123 beats per minute, his blood pressure was 123/85 mm Hg, respiratory rate was 18/min, and oxygen saturation was 95-98% on room air. Physical exam was remarkable only for cachexia. BMI was 15.38 kg/m^2^. Pertinent labs included hemoglobin of 10.1 g/dL, platelet count of 519,000/mm^3^, sodium of 131 mEq/L, potassium of 5.7 mEq/L, blood urea nitrogen (BUN) of 62 mg/dL, creatinine of 4.3 mg/dL, corrected calcium of 11.14 mg/dL, total protein of 10.7 g/dL, and albumin of 2.4 g/dL. Troponin and lipase were unremarkable. Initial EKG showed sinus tachycardia with a heart rate of 118, possible biatrial enlargement, and peaked T-waves. Repeat EKG, after hyperkalemia treatment in the emergency department, showed a rate of 85, normal axis, normal intervals, and no T-wave inversions. Chest X-ray showed no acute cardiopulmonary process. CT of the brain without contrast was normal. In the emergency department, the patient was given 20 mg of furosemide once, 25 g of D5W, five units of regular insulin, 30 mg of ketorolac, one bolus of lactated Ringer, and one bolus of normal saline. The patient was ultimately admitted to the internal medicine department for chronic diffuse myalgias, anemia, several electrolyte abnormalities (Table 1), and acute kidney injury.

**Table 1:** Admission labs

Variable	Reference range, adults	On admission
Hematocrit (%)	36-46	32.5
Hemoglobin (g/dL)	12-16	10.1
White blood cell count (per ul)	4,500-11,000	9,580
Neutrophils	40-70	56.2
Lymphocytes	22-44	17.8
Monocytes	4-11	6.3
Eosinophils	0-8	19
Basophils	0-3	0.3
Platelet count (per ul)	150,000-400,000	519,000
Red blood cell count (per ul)	4,000,000-5,900,000	3,630,000
Mean corpuscular volume (fl)	80-100	89.5
Sodium (mmol/liter)	135-145	131
Potassium (mmol/liter)	3.4-5.0	5.7
Chloride (mmol/liter)	98-108	99
Carbon dioxide (mmol/liter)	23-32	22
Glucose (mg/dl)	70-110	193
Albumin (g/dl)	3.3-5.0	2.7
Total protein (g/dl)	6.0-8.3	9.6

The patient was initially worked up under clinical suspicion of multiple myeloma, due to elevated erythrocyte sedimentation rate at 134 and lack of renal symptoms aside from urinary hesitancy. C-reactive protein was 1.62 mg/L. Urinalysis showed moderate blood, 30 mg/dL of protein, RBC of 10/hpf, and was negative for ketones and glucose. Repeat urinalysis showed hyaline casts at 8/lpf. Upon further workup, serum protein electrophoresis (SPEP) and urine protein electrophoresis (UPEP) were not consistent with monoclonal proteinopathy. Serum flow cytometry showed no immunophenotypic abnormalities identified, and no increase in plasma cells. Moreover, flow cytometry of bone marrow showed no immunophenotypic evidence of acute leukemia, B-cell or T-cell lymphoproliferative disorders, or plasma cell neoplasm. Bone marrow biopsy showed normal cellular marrow with trilineage hematopoietic elements with no evidence of abnormal infiltrates. The bone survey was unremarkable. Further rheumatological inflammatory and autoimmune disease workups were done. Sjögren's antibody, anti-smooth muscle, thyroid-stimulating hormone (TSH) and T4, rheumatoid factor, cyclic citrullinated peptide IgG, alpha fetal protein, cancer antigen 19-9 (CA 19-9), prostate-specific antigen, carcinoembryonic antigen, antinuclear antibodies, human leukocyte antigen B27 (HLA-B27), glomerular basement membrane IgG, thyroid peroxidase antibody, thyroid antithyroglobulin, and anti-proteinase 3 (PR3) were all negative.

**Figure 1:**
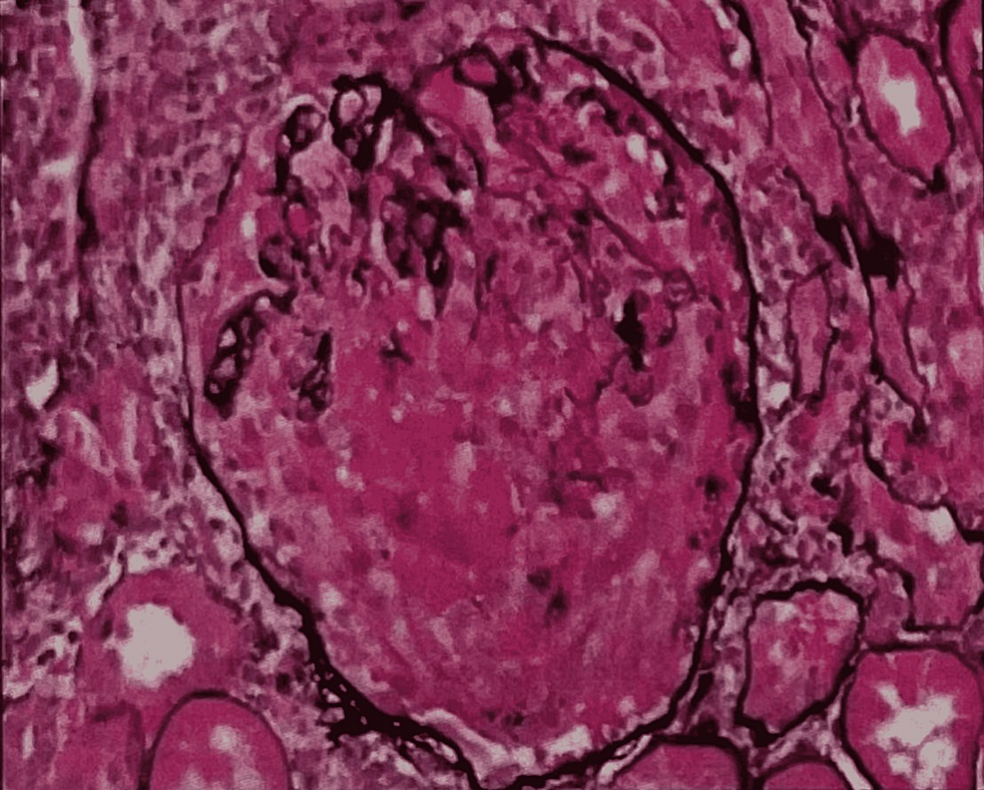
Kidney biopsy on light microscopy Sections hematoxylin and eosin (H&E) x2, periodic acid-Schiff (PAS) x2, and Jones methenamine silver x2 (all prepared at an outside institution) show three pieces of corticomedullary junctions (20-30% cortex) containing up to 10 glomeruli, four of which are globally sclerosed. Additionally, four H&E-stained frozen section slides of tissue for immunofluorescence are reviewed by light microscopy. At least one globally sclerosed glomerulus has an associated fibrous crescent while another corrugation and focal segmental contour duplication without the appearance of spike or holes by silver stains. Three glomeruli have cellular crescents with associated basement membrane breaks and fibrinoid necrosis, while another extensively segmentally sclerosed glomerulus has a fibrocellular crescent, but there is no endocapillary proliferation. There are multifocal lymphoplasmacytic infiltrates with scattered eosinophils and rare neutrophils, as well as associated tubulitis and acute tubular injury characterized by epithelial cell flattening, vacuolization, apical blebbing, sloughing, and loss of brush borders. There is 30-40% interstitial fibrosis and tubular atrophy. There are scattered proteinaceous, occasional granular, and rare blood cell casts, as well as one small focus of intratubular calcium oxalate crystals. Arterioles show moderate medial thickening and there is moderate to focally severe intimal fibrosis and medial thickening of interlobular arteries. One small interlobular artery/arteriole on the edge of the biopsy shows fibrinoid necrosis of the vessel wall with marked inflammation and frequent eosinophils.

**Figure 2:**
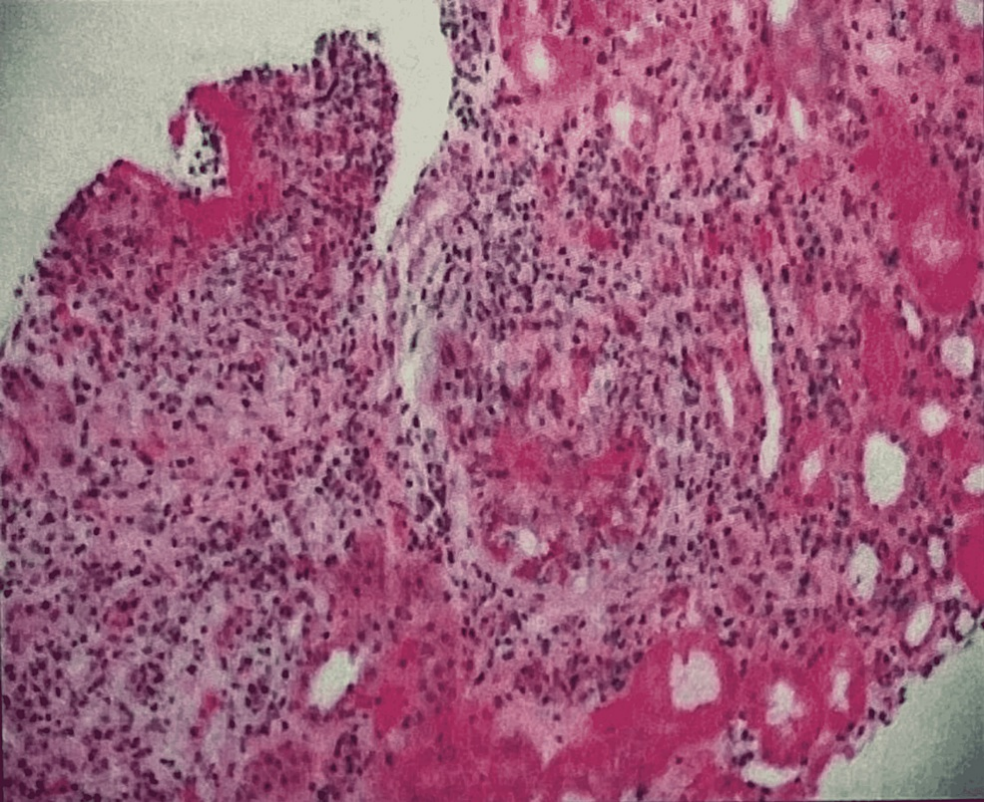
Kidney biopsy on immunofluorescence microscopy Frozen sections containing up to six glomeruli, two of which are globally sclerosed and at least three with basement membrane breaks, are stained with fluorescent antisera to IgG, IgA, IgM, C3, C1q, kappa, lambda, albumin, and fibrinogen. On a scale of 0 to 3+, there is nonspecific segmental granular staining in injured glomeruli for IgG (1+), IgM (trace), C3 (1+), C1q (trace), kappa (1+), and lambda (1), as well as 3+ segmental staining for fibrinogen in glomeruli with basement membrane breaks. Casts stain 3+ for IgA, kappa, and lambda, and there is arteriolar staining for IgM (1+) and C3 (2+). There is focal protein resorption droplet staining for albumin.

**Figure 3:**
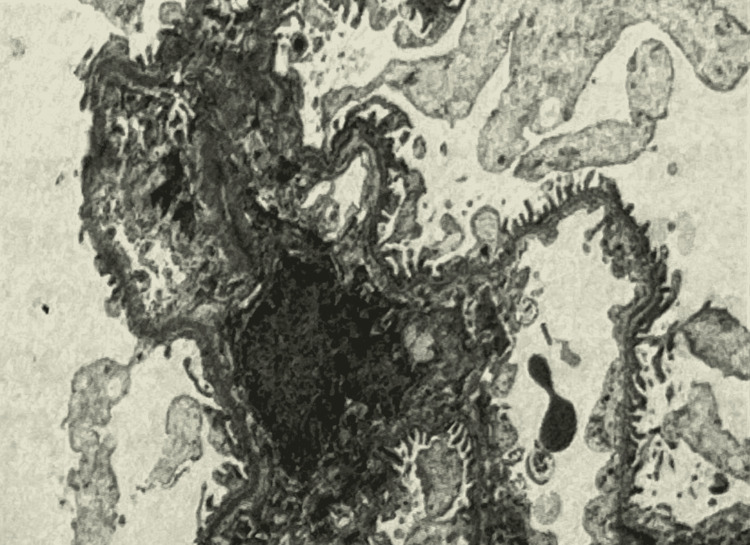
Kidney biopsy on electron microscopy The specimen submitted for electron microscopy, studied first by light microscopic examination of toluidine blue-stained one-micron-thick sections, shows fragments of renal cortex containing two glomeruli, neither of which is globally sclerosed. Morphologic features are similar to those described in light microscopy. Ultrastructural examination reveals portions of capillary loops with patent lumina. Glomerular basement membranes are not significantly thickened. There is a rare (1) intramembranous density, but there are no diagnostic immune complex deposits. There is mild (10-20%) foot process effacement. Occasional inflammatory cells are present within capillary loops. There is no significant increase in mesangial matrix or cellularity. There are no tubular basement membrane deposits.

**Figure 4:**
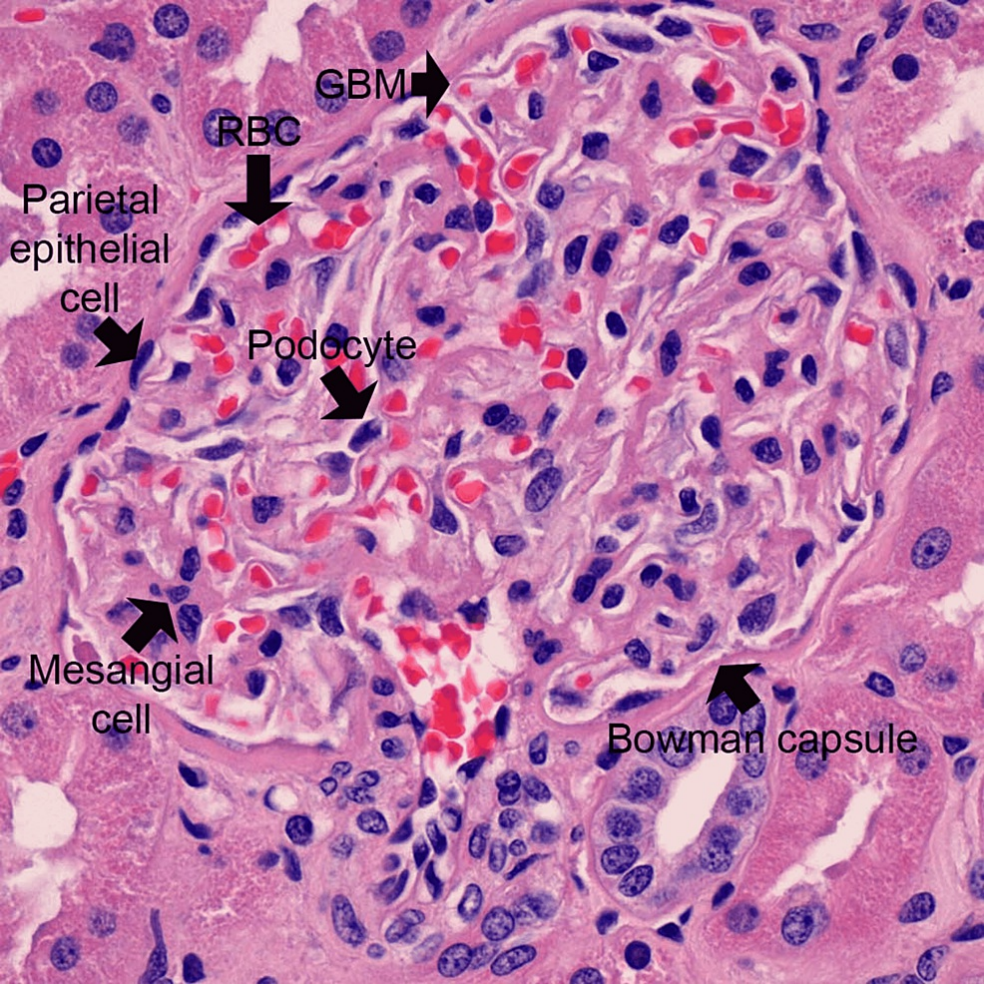
Normal glomerulus on hematoxylin and eosin stain Figure by Anthony Chang, MD, Pathology, University of Chicago. GBM, glomerular basement membrane.

Urine and serum-free kappa and lambda chains quantitative and beta 2 microglobulin were elevated. UPEP showed proteinuria. Cytoplasmic antineutrophil cytoplasmic antibody (c-ANCA) titer was 1:320. Myeloperoxidase (MPO) antibody was elevated at 623.2. Anti-PR3 was negative. Subsequent renal biopsy was obtained and findings were characteristic of pauci-immune NCGN, showing crescents with fibrinoid necrosis and several basement membrane breaks (Figures 1-4). The patient was started on Solu-Medrol 1 g for three days and rituximab 1000 mg one dose every 14 days for two doses. At that point, the patient had been weaned off methylprednisolone 1 g and switched to prednisone 40 mg daily. The patient was additionally started on *Pneumocystis jirovecii* and *Cytomegalovirus* prophylaxis of trimethoprim-sulfamethoxazole and valganciclovir as well as multimodal pain management. Rheumatology and nephrology were consulted throughout the workup of the patient.

Throughout the hospitalization, the patient’s renal markers remained elevated with a BUN ranging from 96 to 98 mg/dL and creatinine stabilized ranging from 3.9 to 4.1 mg/dL. Baseline creatinine was 0.9 mg/dL. The patient continued to have steady urine output but was placed on strict input/output. Additionally, normocytic normochromic anemia was attributed to multifactorial causes including vasculitis and iron deficiency. In terms of his persistent hyponatremia around 130, it was believed this was pseudohyponatremia in the setting of elevated total protein with a protein gap. He was also persistently hyperkalemic and was treated with sodium zirconium cyclosilicate 10 g three times a day (TID). Throughout the course of his hospitalization, he received several doses of calcium gluconate due to elevated potassium (ranging from 5.2 to 5.8 mm/L). EKG was stable throughout hospitalization. Lastly, the patient’s type 2 diabetes was treated with insulin.

## Discussion

The initial presentation of the patient included several months of joint pain, back pain, unintentional weight loss, and electrolyte abnormalities. The patient denied other symptoms that can be expressed in c-ANCA vasculitis. The patient showed no respiratory symptoms (dyspnea, cough, or hemoptysis), no hematuria, and no purpura or urticaria, nausea, vomiting, diarrhea, or abdominal pain. Therefore, the patient was initially worked up under the suspicion of multiple myeloma. The unusual presentation of ANCA vasculitis displayed in this case emphasizes the importance of keeping vasculitis within the differential [2]. Patients suffering from ANCA-positive vasculitis tend to have more defined symptomatology that allows for early detection either at the primary-care level or when an acute event requires hospitalization. The fact that this patient’s vasculitis was able to evade detection for several months is proof that a high index of clinical suspicion is paramount in future diagnostic methods.

The pathogenesis of ANCA vasculitides involves a combination of the environment, genetic predisposition, and responses of the adaptive and natural immune systems. When ANCA autoantibodies concealed in cytoplasmic granules of polymorphonuclear leukocytes (PMN) are revealed to cytokines and proinflammatory mediators, acute vascular inflammation occurs. This leads to the emergence of the ANCA antigens on the surface of the neutrophils and their release. ANCA cohere with the ANCA surface antigens leads to neutrophil activation. These activated neutrophils initiate the alternative complement pathway, which prepares other neutrophils for activation by ANCA via chemoattractant C5a. Activated neutrophils transmigrate across endothelium toward chemoattractants such as C5a and subsequently release oxygen radicals and inflammation-causing destructive enzymes, resulting in necrotizing inflammation of small vessels. Indirect immunofluorescence staining techniques allow for the differentiation of c-ANCA and perinuclear ANCA. Two target antigens of ANCA in AAV are PR3 and MPO. The c-ANCA pattern is mostly caused by antibodies to PR3 but can react with MPO or bactericidal/permeability-increasing protein [3].

ANCA is detected in 90% of cases of pauci-immune renal vasculitis with focal glomerular necrosis and crescent formation [4]. Upon biopsy, this patient was diagnosed with pauci-immune NCGN. The patient had an associated c-ANCA titer of 1:320 but negative PR3. Instead, MPO was elevated at 623.2. Both PR3 ANCA positivity and lung involvement have a strong predictive value for relapse, neither of which was seen in this patient [5]. The patient was subsequently placed on rituximab 1000 mg on days one and 15 due to the severity of the disease. The guidelines for the treatment of AAV according to the American College of Rheumatology/Vasculitis Foundation recommend IV methylprednisolone 500-1000 mg/day (adults) or equivalent for three to five days for initial treatment. Remission induction therapy for active or severe disease consists of rituximab 1000 mg on days one and 15 for adults [6].

## Conclusions

ANCAs are a type of autoantibodies that are associated with necrotizing small-vessel vasculitis collectively known as AAV. AAV includes MPA, GPA, EGPA, and renal-limited NCGN. ANCA vasculitis can present in a myriad of ways, including but not limited to fever, weight loss, arthralgias, myalgias, dyspnea, cough, hemoptysis, hematuria, purpura or urticaria, nausea, vomiting, diarrhea, or abdominal pain. A crucial aspect of the diagnosis of ANCA vasculitis is suspicion, especially when investigating unidentified inflammatory processes. Diagnosis is confirmed by biopsy. However, a negative ANCA does not rule out vasculitis.
